# Supplementary material for: Increased Hippocampal-Inferior Temporal Gyrus White Matter Connectivity following Donepezil Treatment in Patients with Early Alzheimer’s Disease: A Diffusion Tensor Probabilistic Tractography Study
Source: J Clin Med. 2023 Jan 27;12(3):967. doi: 10.3390/jcm12030967 (PMC9917574; doi:10.3390/jcm12030967)
Supplement: Supplementary file 1 [file jcm-12-00967-s001.zip › jcm-2079257-supplementary.pdf]

Increased Hippocampal-Inferior Temporal Gyrus White Matter Connectivity Following Donepezil Treatment in Patients with Early Alzheimer's Disease: A Diffusion Tensor Probabilistic Tractography Study

Supplementary Materials

Supplementary Methods

Participants excluded from the analyses of the current study:

Among the 25 patients with early-stage Alzheimer's disease (AD) who were scanned, 15 were excluded from all analyses (Supplementary Figure S1). After performing the 1st MR examination, 13 were excluded: 11 patients who declined to participate; 2 patients with a structural abnormality detected in the anatomical scan. After performing the 2nd MR examination, 2 patients were excluded because of structural brain abnormalities.

Among the 12 health controls who were scanned, 3 were excluded from all analyses including: 2 subjects with a structural abnormality detected in the anatomical scan; 1 subject who had no diffusion tensor imaging (DTI) data (Supplementary Figure S1).

Voxel-Based Morphometry:

MRI data were analyzed using SPM8 software (Statistical Parametric Mapping, Wellcome Department of Cognitive Neurology, University College, London, U.K.) with diffeomorphic anatomical registration through exponentiated Lie algebra (DARTEL) analysis. Prior to data processing, all individual data were aligned the anterior and posterior commissures line on the transverse plane. After correction of the non-uniformity field bias on images, MRI data were segmented to gray matter (GM), white matter (WM), and cerebrospinal fluid (CSF) using the tissue probability maps based on International Consortium of Brain Mapping (ICBM) space template type of East Asian Brains. The mean templates of GM and WM were created using individual GM and WM images. All the images were normalized to the Montreal Neurological Institute template and were subsequently modulated concerning GM and WM volumes. Then, images smoothed with a 8 mm full width at a half maximum (FWHM) isotropic Gaussian kernel. A Mann-Whitney U-test was used to compare brain volume between healthy controls vs. patients with early AD, and a Wilcoxon signed-rank test was used to compare brain volume between patients treated with and without donepezil using SPSS (version 27.0, IBM, Armonk, NY, USA).

Supplementary Results

Gray and white matter volume changes:

Compared with healthy controls, patients with early AD showed significantly reduced GM volumes in the hippocampus ([x, y, z = 32, −16, −14], t-value = 5.8) (FWE corrected, $p < 0.05$). Whereas, the brain areas with decreased WM volume were not observed in patients with early AD compared with healthy controls. In addition, no significant differences were detected in the WM volumes between patients with early AD and treated patients.

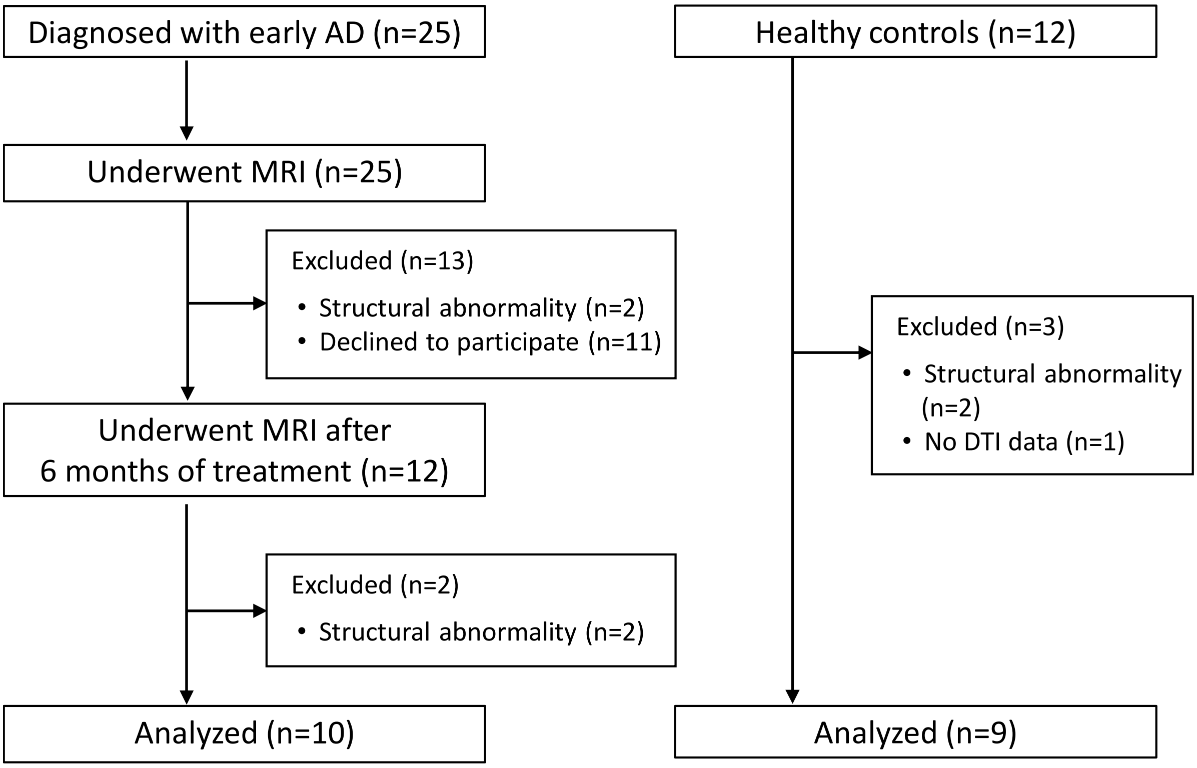

Figure S1. A flow chart showing the cohorts used in the analyses.

Table S1. Demographic and clinical characteristics of patients with AD, donepezil-treated patients, and control.

	MCI patients			Healthy controls (n=9, HC)	Statistical analysis (p-value)		
	Baseline (n=10)	Follow-up (n=10)	Mean rate after treatment		Baseline vs. Follow-up	HC vs. Baseline	HC vs. Follow-up
Age (years)	73.1±7.9	-	-	70.7±3.5	-	p=0.092	p=0.092
Gender	7 F, 3 M	-	-	6 F, 3 M	-	p=0.876	p=0.876
K-MMSE	16.5±4.9	17.5±2.9	+ 7.9%	28.6±1.1	p=0.031[a]	p<0.001[b]	p<0.001[b]
ADAS-Cog	25.6±6.2	24.4±5.9	- 4.7%	-	p=0.506	-	-
CDR	0.6±0.2	0.6±0.2	0%	-	p=0.317	-	-
GDS	13.2±5.2	12.7±4.9	- 3.8%	-	p=0.372	-	-

[a] Significant difference (Wilcoxon's signed-ranks; $p < 0.05$) between early AD patients (baseline) and treated patients (follow-up). [b] Significant differences (Mann-Whitney U; $p < 0.001$) in both "healthy controls (HC) vs. early AD patients" and "HC vs. treated patients". K-MMSE, the Korean version of the mini-mental state examination; ADAS-Cog, AD assessment scale-cognitive subscale; CDR, clinical dementia rating.

Table S2. Differential fractional anisotropy (FA) in the patients with early AD (baseline), donepezil-treated patients (Follow-up), and healthy controls (HC).

Brain areas	MCI patients (Baseline)	Treated patients (Follow-up)	Healthy controls (HC)	Statistical analysis		
				Baseline vs. follow-up	HC vs. baseline	HC vs. follow-up
Amygdala	0.130±0.022	0.130±0.017	0.155±0.032	p=0.508	p=0.009	p=0.018
Caudate nucleus	0.146±0.022	0.154±0.014	0.182±0.026	p=0.646	p=0.327	p=0.211
Hippocampus	0.200±0.024	0.197±0.040	0.218±0.035	p=0.333	p=0.050	p=0.041
Putamen	0.269±0.029	0.270±0.024	0.284±0.030	p=0.878	p=0.253	p=0.253
Thalamus	0.285±0.019	0.291±0.027	0.300±0.018	p=0.203	p=0.253	p=0.624
SFG	0.137±0.013	0.133±0.018	0.130±0.013	p=0.285	p=0.288	p=0.624
MFG	0.140±0.019	0.128±0.021	0.130±0.014	p=0.017	p=0.327	p=0.806
IFG	0.123±0.015	0.121±0.013	0.133±0.015	p=0.575	p=0.191	p=0.072
STG	0.128±0.014	0.126±0.015	0.131±0.020	p=0.721	p=0.806	p=0.870
MTG	0.122±0.017	0.118±0.009	0.127±0.012	p=0.646	p=0.253	p=0.142
ITG	0.160±0.010	0.153±0.009	0.170±0.020	p=0.059	p=0.221	p=0.050

SFG; superior frontal gyrus, MFG; middle frontal gyrus, IFG; inferior frontal gyrus, STG; superior temporal gyrus, MTG; middle temporal gyrus, ITG; inferior temporal gyrus. * significant difference (Bonferroni corrected, $p < 0.05$).

Table S3. Differential mean diffusivity (MD) in the patients with early AD (baseline), donepezil-treated patients (Follow-up), and healthy controls (HC).

Brain areas	MCI patients (Baseline)	Treated patients (Follow-up)	Healthy controls (HC)	Statistical analysis		
				Baseline vs. follow-up	HC vs. baseline	HC vs. follow-up
Amygdala	0.00121±0.00022	0.00121±0.00017	0.00102±0.00004	p=0.878	p=0.003*	p=0.004*
Caudate nucleus	0.00146±0.00019	0.00147±0.00023	0.00129±0.00012	p=0.878	p=0.045	p=0.086
Hippocampus	0.00132±0.00014	0.00133±0.00023	0.00116±0.00009	p=0.221	p=0.041	p=0.165
Putamen	0.00094±0.00018	0.00094±0.00014	0.00086±0.00008	p=0.386	p=0.191	p=0.253
Thalamus	0.00116±0.00020	0.00115±0.00019	0.00107±0.00003	p=0.760	p=0.165	p=0.414
SFG	0.00125±0.00015	0.00124±0.00015	0.00131±0.00013	p=0.799	p=0.191	p=0.165
MFG	0.00129±0.00020	0.00131±0.00020	0.00135±0.00013	p=0.508	p=0.327	p=0.369
IFG	0.00137±0.00013	0.00137±0.00014	0.00131±0.00011	p=0.859	p=0.221	p=0.369
STG	0.00130±0.00015	0.00129±0.00017	0.00124±0.00013	p=0.508	p=0.253	p=0.568
MTG	0.00112±0.00014	0.00111±0.00015	0.00103±0.00008	p=0.878	p=0.165	p=0.205
ITG	0.00095±0.00006	0.00097±0.00007	0.00089±0.00006	p=0.878	p=0.072	p=0.014

SFG; superior frontal gyrus, MFG; middle frontal gyrus, IFG; inferior frontal gyrus, STG; superior temporal gyrus, MTG; middle temporal gyrus, ITG; inferior temporal gyrus. * significant difference (Bonferroni corrected, $p < 0.05$).

Table S4. Differential radial diffusivity (RD) in the patients with early AD (baseline), donepezil-treated patients (Follow-up), and healthy controls (HC).

Brain areas	MCI patients (Baseline)	Treated patients (Follow-up)	Healthy controls (HC)	Statistical analysis		
				Baseline vs. follow-up	HC vs. baseline	HC vs. follow-up
Amygdala	0.00112±0.00022	0.00112±0.00016	0.00094±0.00004	p=0.959	p=0.002*	p=0.002*
Caudate nucleus	0.00135±0.00019	0.00135±0.00023	0.00117±0.00012	p=0.445	p=0.060	p=0.102
Hippocampus	0.00124±0.00012	0.00125±0.00023	0.00108±0.00010	p=0.203	p=0.027	p=0.121
Putamen	0.00081±0.00018	0.00081±0.00013	0.00073±0.00008	p=0.878	p=0.141	p=0.253
Thalamus	0.00101±0.00019	0.00100±0.00019	0.00092±0.00003	p=0.959	p=0.191	p=0.369
SFG	0.00118±0.00015	0.00117±0.00015	0.00125±0.00013	p=0.575	p=0.191	p=0.165
MFG	0.00122±0.00019	0.00125±0.00020	0.00128±0.00012	p=0.575	p=0.307	p=0.414
IFG	0.00131±0.00012	0.00130±0.00014	0.00124±0.00011	p=0.575	p=0.253	p=0.327
STG	0.00123±0.00015	0.00122±0.00017	0.00117±0.00013	p=0.959	p=0.253	p=0.514
MTG	0.00106±0.00014	0.00106±0.00015	0.00098±0.00008	p=0.799	p=0.142	p=0.165
ITG	0.00089±0.00006	0.00091±0.00007	0.00083±0.00006	p=0.285	p=0.060	p=0.014

SFG; superior frontal gyrus, MFG; middle frontal gyrus, IFG; inferior frontal gyrus, STG; superior temporal gyrus, MTG; middle temporal gyrus, ITG; inferior temporal gyrus. * significant difference (Bonferroni corrected, $p < 0.05$).

Table S5. Differential axial diffusivity (AD) in the patients with early AD (baseline), donepezil-treated patients (Follow-up), and healthy controls (HC).

Brain areas	MCI patients (Baseline)	Treated patients (Follow-up)	Healthy controls (HC)	Statistical analysis		
				Baseline vs. follow-up	HC vs. baseline	HC vs. follow-up
Amygdala	0.00137±0.00022	0.00138±0.00019	0.00120±0.00005	p=0.878	p=0.011	p=0.034
Caudate nucleus	0.00170±0.00021	0.00171±0.00025	0.00153±0.00012	p=0.285	p=0.041	p=0.142
Hippocampus	0.00149±0.00016	0.00150±0.00025	0.00134±0.00009	p=0.241	p=0.102	p=0.288
Putamen	0.00120±0.00018	0.00119±0.00016	0.00111±0.00008	p=0.721	p=0.253	p=0.165
Thalamus	0.00146±0.00021	0.00145±0.00020	0.00136±0.00003	p=0.838	p=0.221	p=0.369
SFG	0.00138±0.00016	0.00136±0.00015	0.00144±0.00013	p=0.203	p=0.191	p=0.191
MFG	0.00143±0.00021	0.00144±0.00021	0.00149±0.00014	p=0.959	p=0.414	p=0.369
IFG	0.00151±0.00013	0.00151±0.00016	0.00145±0.00011	p=0.386	p=0.288	p=0.567
STG	0.00143±0.00015	0.00142±0.00017	0.00136±0.00013	p=0.959	p=0.253	p=0.567
MTG	0.00122±0.00014	0.00121±0.00016	0.00114±0.00008	p=0.541	p=0.165	p=0.288
ITG	0.00108±0.00007	0.00108±0.00008	0.00101±0.00006	p=0.507	p=0.086	p=0.050

SFG; superior frontal gyrus, MFG; middle frontal gyrus, IFG; inferior frontal gyrus, STG; superior temporal gyrus, MTG; middle temporal gyrus, ITG; inferior temporal gyrus. * significant difference (Bonferroni corrected, $p < 0.05$).

References

1. Lin, G.A.; Whittington, M.D.; Synnott, P.G.; McKenna, A.; Campbell, J.; Pearson, S.D.; Rind, D.M. *Aducanumab for Alzheimer's Disease: Effectiveness and Value*; Final Evidence Report and Meeting Summary; Institute for Clinical and Economic Review: Available online: Boston, MA, USA, 2021. Available online: https://icer.org/wp-content/uploads/2020/10/ICER_ALZ_Draft_Evidence_Report_050521.pdf (accessed on 4 October 2021).
2. Robinson, R.L.; Rentz, D.M.; Andrews, J.S.; Zagar, A.; Kim, Y.; Bruemmer, V.; Schwartz, R.L.; Ye, W.; Fillit, H.M. Costs of Early Stage Alzheimer's Disease in the United States: Cross-Sectional Analysis of a Prospective Cohort Study (GERAS-US)1. *J. Alzheimers Dis.* **2020**, *75*, 437–450.
3. Assuncao, S.S.; Sperling, R.A.; Ritchie, C.; Kerwin, D.R.; Aisen, P.S.; Lansdall, C.; Atri, A.; Cummings, J. Meaningful benefits: A framework to assess disease-modifying therapies in preclinical and early Alzheimer's disease. *Alzheimers Res. Ther.* **2022**, *14*, 54.
4. Tahami Monfared, A.A.; Houghton, K.; Zhang, Q.; Mauskopf, J.; Alzheimer's Disease Neuroimaging, I. Staging Disease Severity Using the Alzheimer's Disease Composite Score (ADCOMS): A Retrospective Data Analysis. *Neurol. Ther.* **2022**, *11*, 413–434.
5. Seltzer, B.; Zolnouni, P.; Nunez, M.; Goldman, R.; Kumar, D.; Ieni, J.; Richardson, S.; Donepezil "402" Study, G. Efficacy of donepezil in early-stage Alzheimer disease: A randomized placebo-controlled trial. *Arch. Neurol.* **2004**, *61*, 1852–1856.
6. Lai, X.; Wen, H.; Li, Y.; Lu, L.; Tang, C. The Comparative Efficacy of Multiple Interventions for Mild Cognitive Impairment in Alzheimer's Disease: A Bayesian Network Meta-Analysis. *Front Aging Neurosci.* **2020**, *12*, 121.
7. Gaynes, B.N.; Asher, G.; Gartlehner, G.; Hoffman, V.; Green, J. AHRQ Technology Assessments. In *Definition of Treatment-Resistant Depression in the Medicare Population*; Agency for Healthcare Research and Quality: Rockville, MD, USA, 2018.
